# Knockout of IGFBP3 improves alcohol-induced liver injury via Akt/GSK3β and TMEM219/caspase 8 pathways

**DOI:** 10.3389/fphys.2026.1775461

**Published:** 2026-05-15

**Authors:** Huimei Cai, Chenyu Zhu, Xi Zheng, Yiqi Lian, Enqi Tang, Yushan Chen, Wenyu Cao, Ying Sun, Yi Lv

**Affiliations:** 1 Department of Gastroenterology, Affiliated Fuzhou First Hospital of Fujian Medical University, Fuzhou, China; 2 First Affiliated Hospital of Dalian Medical University, Dalian, China; 3 Clinical Research Center for Oral Tissue Deficiency Diseases of Fujian Province, Fujian Key Laboratory of Oral Diseases, Fujian Provincial Engineering Research Center of Oral Biomaterial, School and Hospital of Stomatology, Fujian Medical University, Fuzhou, China; 4 Fujian Key Laboratory of Developmental and Neural Biology, College of Life Science, Fujian Normal University, Fuzhou, China

**Keywords:** Akt, ALD, GSK3β, IGFBP3, TMEM219

## Abstract

Insulin-like growth factor binding proteins (IGFBPs) are critical regulators of hepatic metabolic homeostasis, and accumulating evidence implicates them in liver disease progression. However, their precise roles and regulatory mechanisms in alcoholic liver disease (ALD) remain elusive. In this study, we performed comprehensive analyses of the expression of IGF system proteins both *in vitro* and *in vivo*, and identified a significant downregulation of IGFBP3 in ALD. Subsequently, we overexpressed IGFBP3 or its mutant IGFBP3^GGG^ with an IGF1-binding site mutation in AML12 cells to evaluate its effect on alcohol-induced hepatocyte injury. Our findings revealed that IGFBP3 overexpression exacerbated alcohol-induced lipid accumulation, reactive oxygen species (ROS) generation, and apoptosis in hepatocytes. Compared with the IGFBP3-overexpressing group, the IGFBP3^GGG^-overexpressing group showed no significant difference in lipid accumulation, whereas the levels of ROS and apoptosis were significantly lower. Furthermore, we generated liver-specific IGFBP3 knockout mice (*Alb-cre;Igfbp3^f/f^*), which exhibited significantly attenuated alcoholic liver injury compared to their *Igfbp3^f/f^* littermates following chronic ethanol exposure. Mechanistically, IGFBP3 overexpression activates the Akt/GSK3β and TMEM219/Caspase8 signaling pathways, driving hepatic lipid accumulation and hepatocyte apoptosis. Paradoxically, knockout of IGFBP3 also promotes the phosphorylation of Akt/GSK3β in liver. Collectively, these findings suggest a potential cross-talk between the Akt/GSK3β and TMEM219/Caspase8 signaling pathways, and the downregulation of IGFBP3 in chronic liver disease may represent a self-regulatory mechanism of hepatocytes to resist adversity.

## 1 Introduction

Alcohol consumption is one of the major causes of morbidity and mortality worldwide (Dukic et al., 2023). According to *the Global Status Report on Alcohol and Health 2018* published by the World Health Organization, about 3 million deaths were attributed to alcohol consumption in 2016 (World Health Organization, 2018). Alcoholic liver disease (ALD) is a progressive liver disorder triggered by excessive alcohol consumption, which encompassing alcoholic steatosis, alcoholic hepatitis, alcoholic cirrhosis, and even superimposed hepatocellular carcinoma (Lv et al., 2020). As the earliest stage of ALD, alcoholic steatosis is characterized by lipid accumulation in hepatocytes and is potentially reversible with alcohol abstinence. However, such lipid accumulation sensitizes the liver to xenobiotics (e.g., endotoxins) and accelerates disease progression to alcoholic hepatitis (Zeng et al., 2014). Despite significant progress in elucidating ALD pathogenesis over the past decades, no FDA-approved therapies currently exist (Ghosh Dastidar et al., 2018). Therefore, identifying diagnostic biomarkers for early-stage ALD and developing novel therapeutic agents to effectively treat ALD patients remain urgent clinical priorities.

The insulin-like growth factor (IGF) system represents a complex network encompassing ligands (IGF1 and IGF2), binding proteins (including IGFBP 1-6), and receptors (IGF1R and IGF2R) (Miller et al., 2022). Within this system, IGF1 plays a pivotal role in regulating hepatic metabolism via mechanisms involving protein synthesis, glucose uptake, and lipid modulation, thereby influencing liver function and systemic energy homeostasis (Bonefeld and Moller, 2011). Clinically, reduced serum IGF1 levels correlate with the severity of hepatocellular dysfunction in chronic liver diseases (Wu et al., 2004; Gui et al., 2023). Therapeutic administration of exogenous IGF1 has shown promise in ALD by reversing alcohol-induced suppression of Akt signaling and attenuating disease progression (Zeng et al., 2018; Luo et al., 2021). IGFBP3 is the primary IGF1-binding protein in the circulation; it can prolong IGF1 half-life and thereby modulate the bioavailability of IGF1 for interaction with IGF receptors, a process critical for regulating IGF1 activity (Lee et al., 2011; Runchey et al., 2014). Emerging evidence indicates that IGFBP3 can also exert biological functions through IGF1-independent mechanisms. For instance, IGFBP3 can induce cellular apoptosis via direct engagement with the transmembrane receptor TMEM219, a mechanism independent of IGF1 (D'addio et al., 2023). The functional versatility of IGFBP3 extends to its involvement in cell proliferation, apoptosis, and migration, with context-dependent effects observed across diverse pathological settings (Han et al., 2015; Bao et al., 2016; Mayo et al., 2017; Wang et al., 2017). In hepatic ischemia-reperfusion injury, hepatic IGFBP3 expression is markedly upregulated, which correlates with increased reactive oxygen species (ROS) production, inflammatory cytokine release, and apoptosis induction (Zhou et al., 2015). In liver fibrosis, IGFBP3 critically promotes hepatic stellate cell activation and migration via the integrin-Akt signaling pathway. *Igfbp3* knockout in mice markedly attenuates carbon tetrachloride (CCl_4_)-induced fibrosis (Yaqoob et al., 2020). Clinical studies have identified a correlation between *Igfbp3* promoter polymorphisms and the incidence of hepatic steatosis (Mahmoudi et al., 2023). Meanwhile, numerous clinical studies have found that the IGFBP3 expression is downregulated in various chronic liver diseases, including ALD (Huang et al., 2022), non-alcoholic fatty liver disease (NAFLD) (Chishima et al., 2017), hepatic fibrosis (Gui et al., 2023) and cirrhosis (Wu et al., 2004), suggesting its potential as a biomarker for chronic liver diseases. However, the specific role and regulatory mechanisms of IGFBP3 in ALD remain poorly characterized.

In the present study, we hypothesized that IGFBP3 may mediate the progression of ALD through dual IGF1-dependent and IGF1-independent pathways. To test this hypothesis, we performed a series of functional studies by overexpressing IGFBP3 and its IGF1-binding deficient mutant IGFBP3^GGG^ in AML12 hepatocytes. Additionally, we utilized liver-specific *Igfbp3* knockout mice to further validate the functional role of IGFBP3 in ALD pathogenesis. Mechanistically, we assessed the regulation of the AKT/GSK3β and TMEM219/Caspase 8 signaling cascades, two critical downstream pathways of IGFBP3. Collectively, this study advances our understanding of the functional role of IGFBP3 in ALD and identifies IGFBP3 as a promising therapeutic target for ALD intervention.

## 2 Materials and methods

### 2.1 Chemical and reagents

Pure ethanol was obtained from Guangzhou Chemical Reagent Factory (Guangzhou, China). Primary antibodies targeting specific proteins were sourced as follows: anti-p-Akt308 (#13038), anti-p-Akt473 (#4060), anti-Akt (#4691), anti-p-GSK-3β (#5558), anti-GSK-3β (#12456), anti-Caspase 8 (#8592), and anti-GAPDH (#97166) antibodies were purchased from Cell Signaling Technology (Beverly, MA, USA); anti-IGFBP3 antibody (#ABS131745) was acquired from Absin (Shanghai, China); and anti-TMEM219 antibody (#AF7556) was obtained from R&D Systems Inc. (Minnesota, USA).

### 2.2 Plasmid construction and transfection

For constructing the IGFBP3 overexpression vector, the coding sequence (CDS) of IGFBP3 was cloned into the pAAV-MCS plasmid, yielding the recombinant plasmid pAAV-MCS-IGFBP3. Meanwhile, the IGFBP3^GGG^ mutant plasmid (designated as pAAV-MCS-IGFBP3^GGG^) was generated following established protocols (Nguyen et al., 2015; Zhou et al., 2015). Subsequently, the transfection mixture was delivered into cells using Lipofectamine™ 2000 according to the manufacturer’s specifications (Thermo Fisher Scientific, USA).

### 2.3 Cell culture and treatment

The murine hepatocyte cell line AML12 was purchased from the Cell Bank of the Type Culture Collection of the Chinese Academy of Sciences (Shanghai, China). Cells were maintained in a humidified incubator with 5% CO_2_ at 37 °C. The culture medium consisted of DMEM/F12 supplemented with 10% fetal bovine serum (FBS), 40 ng/mL dexamethasone, and insulin-transferrin-selenium (ITS). For experimental manipulation, cells were transfected with the indicated plasmid constructs, followed by exposure to 200 mM ethanol for 24 h (Xie et al., 2022).

### 2.4 Animal experiments

Male C57BL/6 mice (6–8 weeks old) were obtained from Shanghai SLAC Laboratory Animal Center (Shanghai, China). *Alb-Cre* and *Igfbp3^f/f^* transgenic mouse strains were purchased from Cyagen Biosciences Inc. (Suzhou, China). Liver-specific Igfbp3 knockout mice (*Alb-Cre; Igfbp3^f/f^*) used in this study were generated by crossing the two parental strains. Six mice were included per experimental group. All mice were housed in a climate-controlled vivarium under standard conditions: temperature 22 ± 2 °C, humidity 50–60%, and a 12-h light/dark cycle. Alcoholic liver disease (ALD) was induced according to the NIAAA model protocol (Bertola et al., 2013). Following euthanasia by rapid cervical dislocation, liver tissues and serum samples were collected for subsequent experiments and analyses. All animal care and experimental procedures were approved by the Institutional Animal Care and Use Committee (IACUC) of Fujian Normal University (Protocol No. IACUC−20190020).

### 2.5 Cell viability assay

Cell viability was assessed using the Cell Counting Kit-8 (#C0038, Beyotime Biotechnology, Shanghai, China) following the manufacturer’s protocol. Briefly, AML12 cells were seeded in 96-well plates at 5×10³ cells/well and cultured overnight under standard conditions for adherence. Following 24-h alcohol treatment, 10 μL of CCK-8 reagent was added to each well, and plates were incubated for another 2 h at 37 °C in the dark. Absorbance was measured at a wavelength of 450 nm using a Multis microplate reader (Thermo Fisher Scientific, USA).

### 2.6 ELISA

The concentrations of IGF-1 and IGFBP3 in cell culture supernatants were determined using commercially available enzyme-linked immunosorbent assay (ELISA) kits (#EM0094 and #EM0098, FineTest, Wuhan, China), according to the manufacturer’s instructions. The absorbance was measured at 450 nm using a microplate reader (Thermo, USA). The concentrations of IGF-1 and IGFBP3 in the samples were calculated by interpolating from the respective standard curves.

### 2.7 Nile red and oil red O staining

Cells or 5-μm-thick frozen liver sections were fixed in 10% neutral buffered formalin at room temperature for 5 min, followed by staining with either Nile Red or Oil Red O solution. Samples were subsequently counterstained with hematoxylin or DAPI, then examined and imaged under a Nikon microscope (Tokyo, Japan).

### 2.8 Biochemical measurement

Serum biochemical parameters, including aspartate aminotransferase (AST), alanine aminotransferase (ALT), total cholesterol (TCHO), triglycerides (TG), low-density lipoprotein cholesterol (LDL-C), high-density lipoprotein cholesterol (HDL-C), and alkaline phosphatase (ALP), were quantitatively determined using the Hitachi LABOSPECT008 automated clinical chemistry analyzer (Hitachi High-Tech Co., Japan).

### 2.9 Flow cytometer analysis

Following cellular treatment, apoptosis was assessed using the Annexin V-FITC Apoptosis Detection Kit (#C1062, Beyotime Biotechnology, Shanghai, China), whereas intracellular reactive oxygen species (ROS) production was quantified with the ROS Assay Kit (#S0033S, Beyotime Biotechnology, Shanghai, China). Both staining protocols were performed strictly according to the manufacturers’ instructions. Subsequently, stained cells were analyzed via flow cytometry on a BD Accuri flow cytometer (BD Biosciences, USA). Data acquisition was set to collect at least 10, 000 events per sample to ensure statistical reliability.

### 2.10 Western blot

Total protein extracts from cells or liver tissues were prepared using RIPA buffer supplemented with a complete EDTA-free protease inhibitor cocktail. The protein lysates were resolved by 10% SDS-PAGE and subsequently transferred onto 0.22 μm nitrocellulose (NC) membranes. Following blocking, the membranes were probed with specific primary antibodies, followed by fluorescence-conjugated secondary antibodies. The relative densities of immunoreactive bands were quantified using the Chemi-Doc XRS imaging system (Bio-Rad, USA) and normalized to the expression level of GAPDH.

### 2.11 Total RNA isolation and quantitative real-time PCR

Total RNA was isolated from AML12 cells or frozen liver tissues using RNAiso Reagent (Takara Bio Inc., Shiga, Japan). First-strand cDNA synthesis was carried out with the PrimeScript™ RT Reagent Kit (Takara Bio Inc., Shiga, Japan) in accordance with the manufacturer’s instructions. Subsequently, gene-specific primers were used to quantify the mRNA expression levels of target genes via quantitative real-time polymerase chain reaction (qRT-PCR) with the Takara SYBR Premix Taq System (Takara Bio Inc., Shiga, Japan) using GAPDH as the internal reference gene (**Table 1**).

**Table 1:** List of primer sequences used for RT-PCR analysis in this study.

Name	Forward	Reverse
IGF1	5’-GCTCTTCAGTTCGTGTGTGGA-3’	5’-GCCTCCTTAGATCACAGCTCC-3’
IGF2	5’-GTGGCATCGTTGAGGAGTG-3’	5’-CACGTCCCTCTCGGACTTG-3’
IGF1R	5’-TCGACATCCGCAACGACTATC-3’	5’-CCAGGGCGTAGTTGTAGAAGAG-3’
IGF2R	5’-CAGAGCGGAGGTTCATCCTAT-3’	5’-CGAATATCGGAGGGTCTGATTGT-3’
IGFBP1	5’-TTGGGACGCCATCAGTACCTA-3’	5’-TTGGCTAAACTCTCTACGACTCT-3’
IGFBP2	5’-GACAATGGCGATGACCACTCA-3’	5’-CAGCTCCTTCATACCCGACTT-3’
IGFBP3	5’-AGACACACTGAATCACCTGAAGT-3’	5’-AGGGCGACACTGCTTTTTCTT-3’
IGFBP4	5’-GGTGACCACCCCAACAACAG-3’	5’-GAATTTTGGCGAAGTGCTTCTG-3’
IGFBP5	5’-ACCTGAGATGAGACAGGAGTC-3’	5’-GTAGAATCCTTTGCGGTCACAA-3’
IGFBP6	5’-GAGGGGCTCAAACACTCTACG-3’	5’-CCATCCGATCCACACACCA-3’
GAPDH	5’-AGGTCGGTGTGAACGGATTTG-3’	5’-TGTAGACCATGTAGTTGAGGTCA-3’

### 2.12 Immunofluorescence

Paraffin-embedded liver tissue sections (5 μm thick) were deparaffinized, rehydrated sequentially, and subjected to antigen retrieval in citrate buffer (pH 6.0). After blocking with 5% bovine serum albumin (BSA) for 1 h at room temperature, sections were incubated overnight at 4 °C with primary antibodies against target proteins. Subsequently, sections were incubated with fluorochrome-conjugated secondary antibodies, followed by nuclear counterstaining with 4’, 6-diamidino-2-phenylindole (DAPI). Fluorescent images were captured using a Zeiss LSM980 confocal laser scanning microscope (CLSM).

### 2.13 Statistical analysis

All data are presented as mean ± SEM. Normality of data distribution was assessed using the Shapiro-Wilk test, and all data met the assumption of normality. Intergroup comparisons were conducted using Student’s t-test or one-way ANOVA, as appropriate. For one-way ANOVA, when a significant difference was indicated (p < 0.05), post-hoc comparisons were performed using Tukey’s multiple comparisons test, which corrects for multiple testing. All analyses were performed using GraphPad Prism 7 software (San Diego, CA). Statistical significance was defined as *p* < 0.05.

## 3 Results

### 3.1 Alcohol induces IGFBP3 downregulation in hepatocytes *in vitro* and *in vivo*

**Figure 1:**
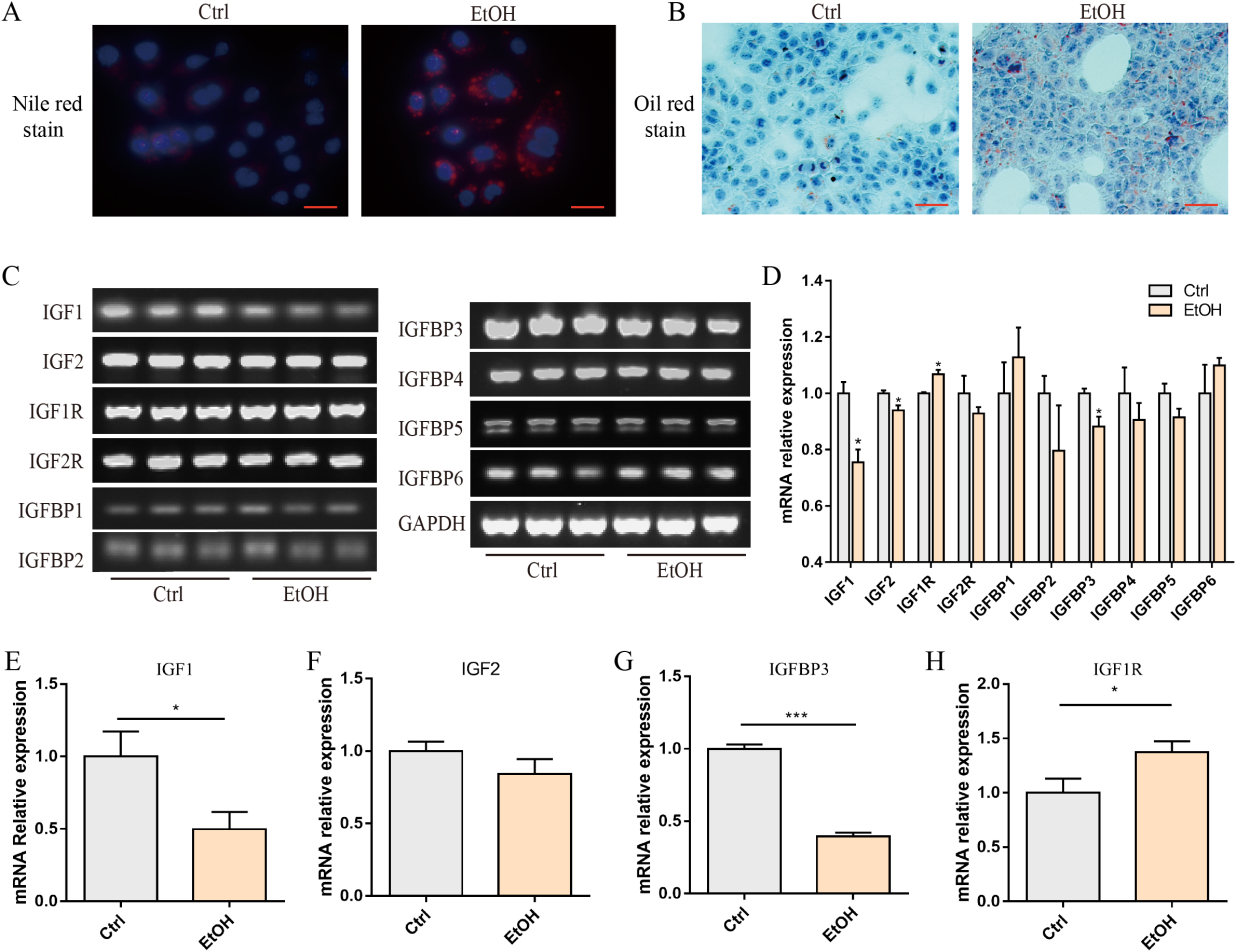
Alcohol inhibited IGFBP3 expression in AML12 cells **(A)** Nile red staining. **(B)** oil red O staining. **(C)** semi-quantitative PCR shows expression patterns of IGF family members. **(D)** densitometric quantification of IGF family member expression. qPCR analysis of mRNA expression: **(E)** IGF1; **(F)** IGF2; **(G)** IGFBP3; **(H)** IGF1R. These data represent mean ± SEM (n=3). Scale bar=200 μm; *p<0.05, ***p < 0.001.

The IGF signaling axis serves as a critical regulator of hepatic energy homeostasis. To systematically investigate the impact of ethanol exposure on the transcriptional landscape of IGF system components in hepatocytes, we established an *in vitro* ALD model using AML12 murine hepatocytes treated with 200 mM ethanol for 24 hours. Following ethanol exposure, a pronounced accumulation of intracellular lipids was observed, as evidenced by the enhanced fluorescence intensities of Nile Red staining and the increased staining density of Oil Red O staining (**Figures 1A, B**). To characterize the gene expression profiles of the IGF system, we performed semi-quantitative reverse transcription-PCR (RT-PCR) analysis coupled with agarose gel electrophoresis. The experimental results demonstrated that following ethanol treatment, the expression of IGF1, IGF2, and IGFBP3 in AML12 cells was significantly downregulated, while IGF1R expression was upregulated. No significant differences were observed in other family members (**Figures 1C, D**). The expression patterns of these four differentially expressed genes were subsequently validated by quantitative real-time PCR (qPCR) (**Figures 1E–H**).

**Figure 2:**
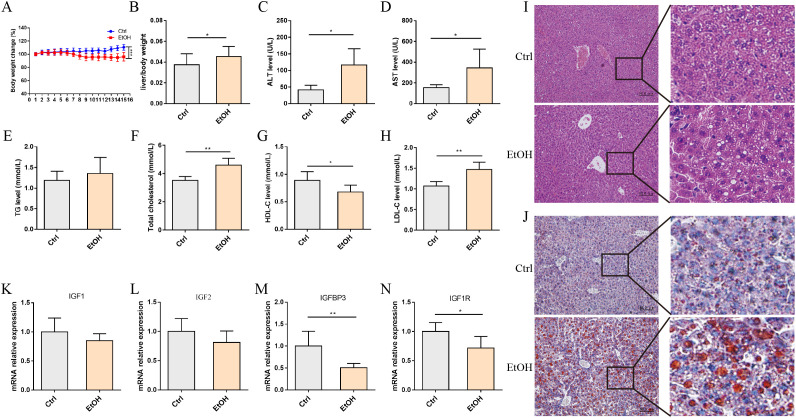
Alcohol feeding inhibited IGFBP3 expression in mouse liver. **(A)** changes in body weight of mice. **(B)** liver/body weight index of mice. Measurement of serum biochemical parameters in mice: **(C)** ALT; **(D)** AST; **(E)** TG; **(F)** TCHO; **(G)** HDL-C; **(H)** LDL-C. **(I)** HE staining; **(J)** oil red O staining. qPCR analysis of mRNA expression: **(K)** IGF1; **(L)** IGF2; **(M)** IGFBP3; **(N)** IGF1R.; scale bar=50 μm. These data represent mean ± SEM (n=6). *p<0.05, **p<0.01, ***p<0.001.

To extend these observations to an *in vivo* context, we employed the Gao-binge ethanol feeding paradigm to induce ALD in C57BL/6J mice (Bertola et al., 2013). Following chronic ethanol administration coupled with a single acute binge dose, experimental animals exhibited significant reductions in body weight concomitant with hepatomegaly, as evidenced by elevated liver-to-body weight ratios compared to pair-fed controls (**Figures 2A, B**). Serum biochemical analysis revealed marked elevations in alanine aminotransferase (ALT) and aspartate aminotransferase (AST) levels that are consistent with previous reports (**Figures 2C, D**). The levels of triglycerides (TG), total cholesterol (TCHO), high-density lipoprotein cholesterol (HDL-C), and low-density lipoprotein cholesterol (LDL-C) in serum were also measured. The results showed a slight increase in TG, while TCHO, LDL-C and HDL-C levels were decreased, suggesting ethanol disrupts hepatic lipid metabolic homeostasis (**Figures 2E–H**). Histological examination using hematoxylin-eosin (HE) and Oil Red O staining confirmed the development of steatosis in ethanol-fed mice (**Figures 2I, J**). Subsequent hepatic qPCR analysis demonstrated significant downregulation of IGF1, IGF2, and IGF1R transcripts, with IGFBP3 exhibiting the most pronounced suppression compared to the control group (**Figures 2K–N**). The conserved IGFBP3 dysregulation pattern observed across both *in vitro* and *in vivo* models, which is consistent with prior clinical reports (Huang et al., 2022), underscores a specific susceptibility of IGFBP3 to ethanol-induced hepatic metabolic stress. Of note, while ethanol upregulated IGF1R expression in AML12 cells (**Figure 1H**), it downregulated IGF1R in mouse livers (**Figure 2N**). This discrepancy may reflect differences between acute *in vitro* exposure and chronic *in vivo* adaptation, and warrants further investigation.

### 3.2 Overexpression of IGFBP3 enhances ethanol-mediated toxicity in AML12 cells

**Figure 3:**
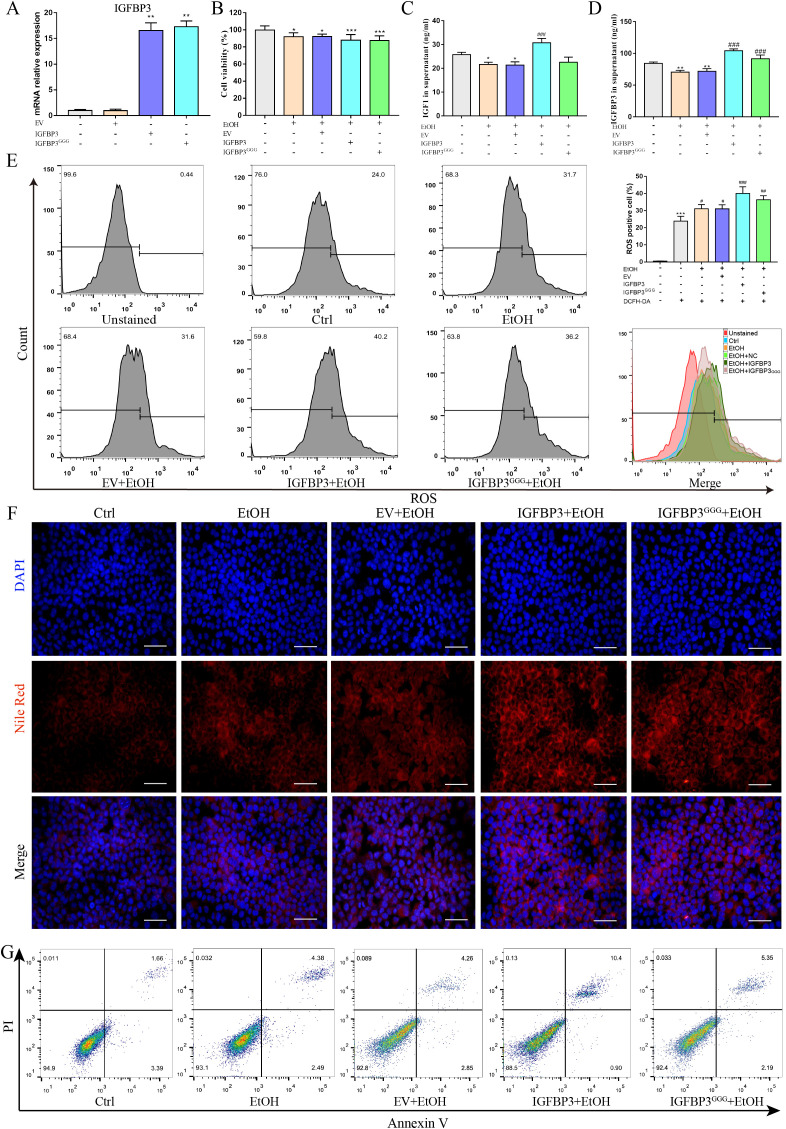
Overexpression of IGFBP3 or IGFBP3^GGG^ *in vitro* promoted alcohol-induced hepatocyte injury. **(A)** quantitative real-time PCR (qPCR) analysis of IGFBP3 mRNA expression. **(B)** cell viability assessment using CCK-8 assay. **(C, D)** ELISA assay determination of IGF1 and IGFBP3 levels in the supernatant. **(E)** flow cytometry-based quantification of ROS levels in AML12 cells. **(F)** Nile red staining. **(G)** flow cytometric analysis of apoptosis in AML12 cells. These data represent mean ± SEM (n=3). EV, empty vector; scale bar=200 μm; *p<0.05, **p<0.01, ***p<0.001 vs. control group; ^#^p<0.05, ^##^p<0.01, ^###^p<0.001, vs. EtOH group.

To elucidate the role of IGFBP3 in ethanol-induced hepatocyte injury, we overexpressed IGFBP3 and its non-IGF-binding mutant variant, IGFBP3^GGG^, in AML12 cells. Following transfection with pAAV-MCS-IGFBP3 and pAAV-MCS-IGFBP3^GGG^ vectors, quantitative real-time PCR was performed to quantify IGFBP3 mRNA expression levels. The results demonstrated a marked upregulation of IGFBP3 mRNA in both overexpression groups relative to the control group (**Figure 3A**). Subsequently, we assessed the impact of IGFBP3 or IGFBP3^GGG^ overexpression on established indicators of ethanol-induced hepatocyte injury, including cell viability, reactive oxygen species (ROS) accumulation, lipid droplet deposition, and apoptotic rate. CCK-8 assays revealed that ethanol exposure significantly impaired the viability of AML12 cells, and this inhibitory effect was further exacerbated by the overexpression of either IGFBP3 or IGFBP3^GGG^ (**Figure 3B**). ELISA assays revealed that ethanol exposure significantly reduced the levels of both IGF−1 and IGFBP3 in the cell culture supernatant. Overexpression of IGFBP3 elevated the concentration of IGF−1 in the supernatant, whereas overexpression of IGFBP3^^GGG^ had no significant effect on IGF−1 levels (**Figures 3C, D**). To measure intracellular ROS levels, we utilized the fluorescent probe DCFH-DA and quantified fluorescence intensity via flow cytometry. Flow cytometric analysis indicated that overexpression of IGFBP3 or IGFBP3^GGG^ both augmented ethanol-induced ROS production. Notably, IGFBP3 overexpression exerted a more pronounced effect than IGFBP3^GGG^ overexpression (positive rates: 40.2% vs. 36.2%, respectively) (**Figure 3E**). Nile Red staining showed that overexpression of either IGFBP3 or IGFBP3^GGG^ promoted ethanol-induced lipid accumulation in AML12 cells, with no statistically significant difference observed between the wild-type and mutant overexpression groups (**Figure 3F**). Using Annexin V-FITC/PI double staining coupled with flow cytometry, we analyzed cellular apoptosis. The results showed that IGFBP3 overexpression significantly enhanced ethanol-induced apoptosis, whereas IGFBP3^GGG^ overexpression only caused a marginal, statistically insignificant increase in apoptotic cells (**Figure 3G**). Collectively, these findings indicate that IGFBP3 overexpression is associated with enhanced ethanol-mediated toxicity, while its non-IGF-binding mutant IGFBP3^GGG^ shows a partial effect.

### 3.3 IGFBP3 activates the Akt/GSK3β and TMEM219/Caspase 8 signaling pathways

**Figure 4:**
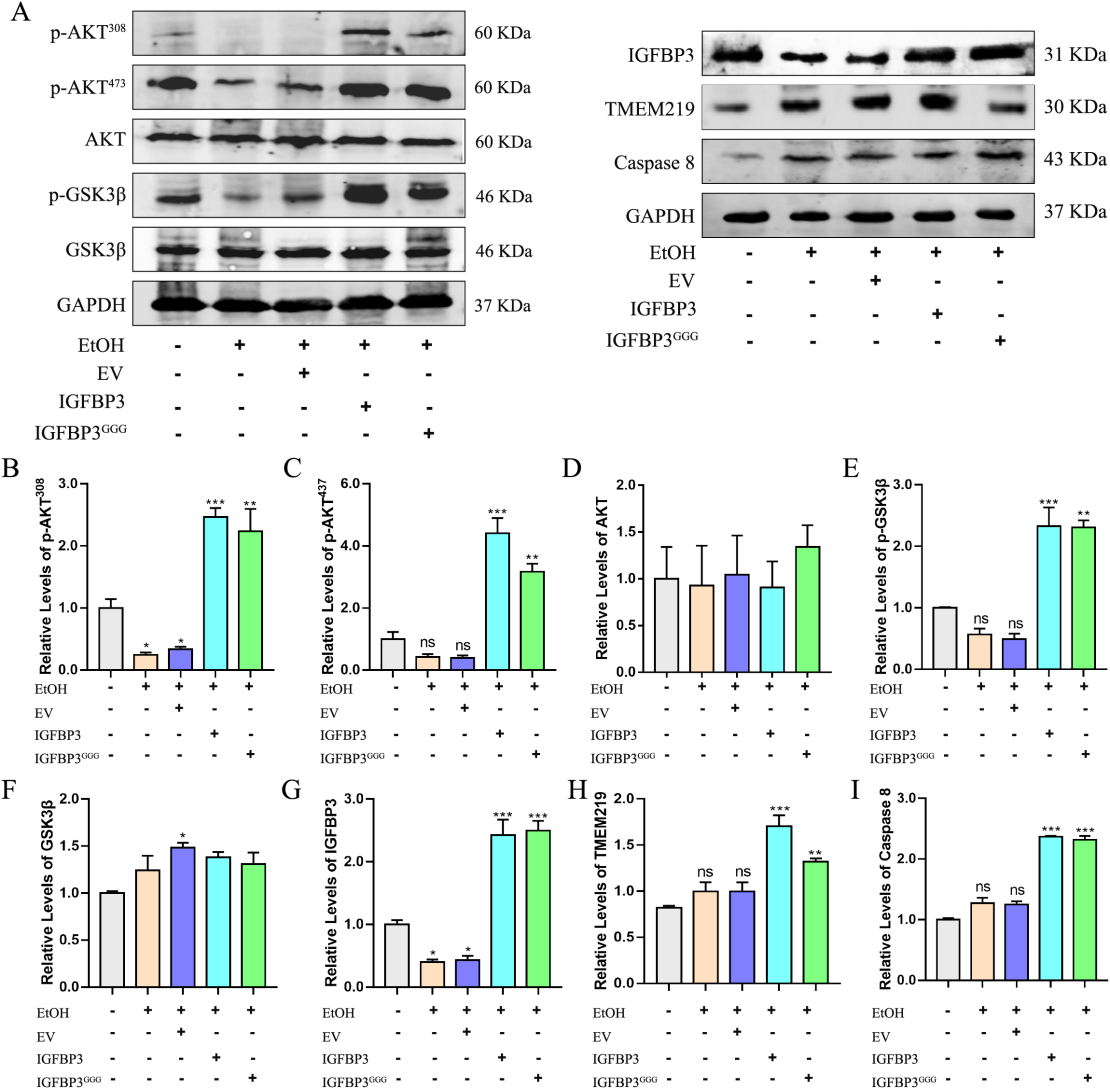
Akt/GSK3β and TMEM219/Caspase 8 signaling in AML12 cells. **(A)** western blot representing Akt/GSK3β and TMEM219/Caspase 8 signaling in AML12 after ethanol-treatment 24 hours. Statistical analysis of protein expression levels: **(B)** p-AKT^308^; **(C)** p-AKT^437^; **(D)** AKT; **(E)** p-GSK3β; **(F)** GSK3β; **(G)** IGFBP3; **(H)** TMEM219; **(I)** Caspase 8. These data represent mean ± SEM (n=3). EV, empty vector; *p<0.05, **p<0.01, ***p<0.001.

Protein kinase B (PKB/Akt) serves as a critical regulator of lipid homeostasis, and its functional dysregulation has been implicated in the pathogenesis of ALD (Wang et al., 2023). Previous studies have shown that ethanol activates hepatic cytochrome P450 2E1 (CYP2E1), inducing oxidative stress in hepatocytes, which in turn suppresses Akt activation. Conversely, IGF1 treatment has been demonstrated to stimulate Akt phosphorylation and attenuate the progression of alcoholic liver disease (Zeng et al., 2018). To investigate whether IGFBP3 could affect Akt activity in an IGF1-dependent manner, we examined the expression and phosphorylation levels of AKT and its downstream effector GSK3β. Experimental results revealed that ethanol reduced the protein levels of p-Akt^308^ and p-Akt^473^ in AML12 cells, as well as those of their downstream target protein p-GSK3β, which is consistent with previous reports (Sun et al., 2018). Surprisingly, overexpression of both IGFBP3 and IGFBP3^GGG^ significantly activated the Akt/GSK3β signaling pathway, which may imply that IGFBP3 can activate the AKT signaling pathway in an IGF1-independent manner (**Figures 4A–F**). Conversely, the downregulation of IGFBP3 in alcohol-exposed hepatocytes may serve to increase free IGF1 levels, representing a self-protective mechanism by which cells resist adverse environmental stress.

Transmembrane Protein 219 (TMEM219), a recently identified receptor for IGFBP3, constitutes a novel component of the cell death signaling pathway. It specifically binds to IGFBP3, and upon activation, propagates apoptotic signals through the downstream Caspase-8 signaling cascade (Baxter, 2013). To explore the role of IGFBP3 in IGF1-independent pathways during ALD progression, we assessed the effects of IGFBP3 and IGFBP3^GGG^ overexpression on the TMEM219/Caspase-8 signaling pathway. Our experimental data showed that ethanol treatment significantly upregulated the protein expression levels of TMEM219 and Caspase-8. Importantly, overexpression of both IGFBP3 and its mutant variant IGFBP3^GGG^ further enhanced the activation of the TMEM219/Caspase-8 signaling cascade (**Figures 4A, G–I**). These results are consistent with previous reports indicating that inhibition of Caspase-8 fails to protect against alcohol-induced liver apoptosis but mitigates alcoholic hepatic steatosis in mice (Hao et al., 2017). Collectively, these findings suggest that IGFBP3 expression is associated with concurrent modulation of the Akt/GSK3β and TMEM219/Caspase-8 pathways, both implicated in ALD pathogenesis.

### 3.4 Liver-specific IGFBP3 knockout alleviates the progression of ALD

**Figure 5:**
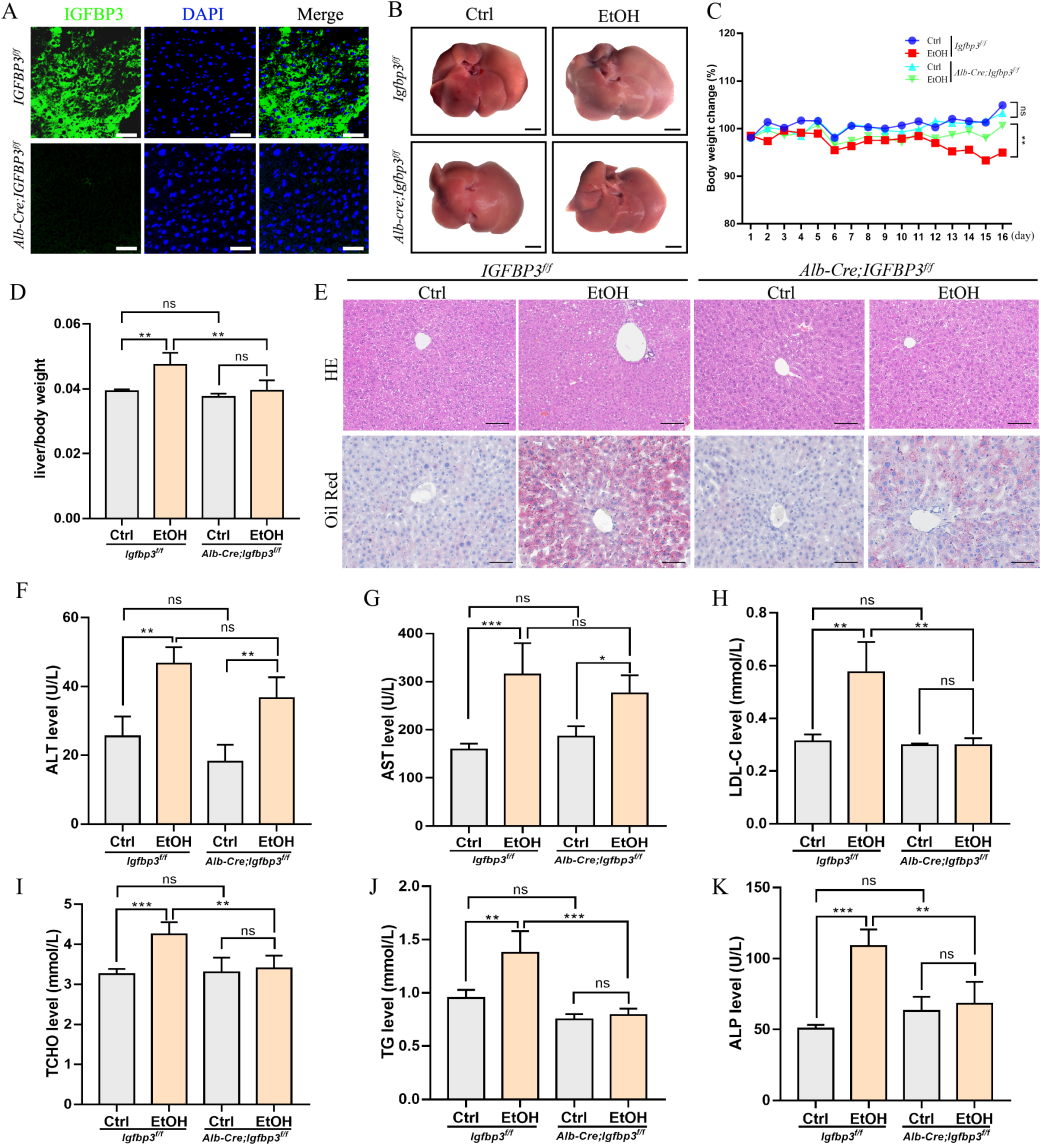
IGFBP3 knockout ameliorated alcoholic liver disease in mice **(A)** immunofluorescence detection of IGFBP3 expression in the livers of *Igfbp3^f/f^* and *Alb-cre;Igfbp3^f/f^* mice. Scale bar=200 μm **(B)** gross liver morphology, scale bar=5 mm. **(C)** changes in body weight of mice. **(D)** liver/body weight index of mice. **(E)** HE and oil red O staining; scale bar=200 μm. Measurement of serum biochemical parameters in mice: **(F)** ALT; **(G)** AST; **(H)** LDL-C; **(I)** TCHO; **(J)** TG; **(K)** ALP. These data represent mean ± SEM (n=6). *p<0.05, **p<0.01, ***p<0.001.

In chronic liver disease, decreased IGFBP3 expression levels may be associated with the maintenance of systemic free IGF1 levels (Yan et al., 2024; Martin-Rivada et al., 2025). To gain deeper insights into the role of reduced IGFBP3 expression in ALD, we generated a liver-specific knockout mouse model, *Alb-cre;Igfbp3^f/f^* (**Figure 5A**). Following ALD induction, alcohol was found to induce hepatomegaly and pale coloration in *Igfbp3^f/f^* mice, whereas the livers of *Alb-cre;Igfbp3^f/f^* mice were less affected by alcohol exposure (**Figure 5B**). Monitoring of body weight and liver-to-body weight ratio revealed that IGFBP3 knockout alleviated the adverse effects of alcohol on mouse body weight and liver weight (**Figures 5C, D**). HE and Oil Red O staining demonstrated a marked reduction in hepatic lipid accumulation in alcohol-fed *Alb-cre;Igfbp3^f/f^* mice compared to alcohol-fed *Igfbp3^f/f^* mice (**Figure 5E**). Biochemical analyses showed that IGFBP3 knockout mitigated alcohol-induced abnormalities in serum-related parameters, including decreased levels of alanine aminotransferase (ALT), aspartate aminotransferase (AST), low-density lipoprotein cholesterol (LDL-C), total cholesterol (TCHO), triglycerides (TG), and alkaline phosphatase (ALP) (**Figures 5F–K**).

### 3.5 IGFBP3 knockout activates Akt/GSK3β but inhibits TMEM219/Caspase8 signaling pathways

**Figure 6:**
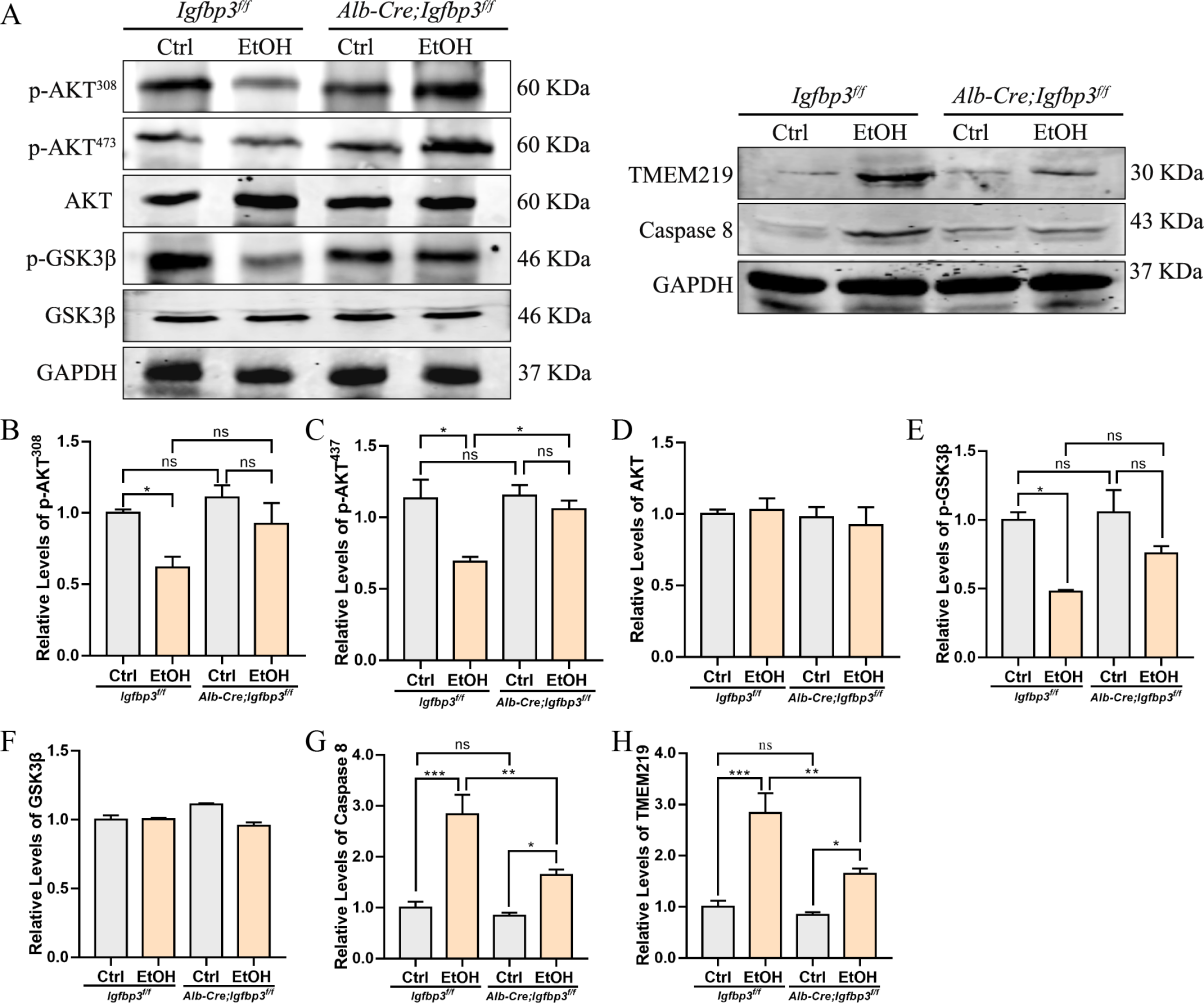
Akt/GSK3β and TMEM219/Caspase 8 signaling in liver. **(A)** western blot representing Akt/GSK3β and TMEM219/Caspase 8 signaling in liver. Statistical analysis of protein expression levels: **(B)** p-AKT^308^; **(C)** p-AKT^437^; **(D)** AKT; **(E)** p-GSK3β; **(F)** GSK3β; **(G)** TMEM219; **(H)** Caspase 8. These data represent mean ± SEM (n=3). *p<0.05, **p<0.01, ***p<0.001.

To gain deeper insights into the effects of IGFBP3 knockout on both IGF-1-dependent and -independent pathways, we examined the expression of proteins associated with the Akt/GSK3β and TMEM219/Caspase 8 signaling pathways. Western blot analysis demonstrated that IGFBP3 knockout reversed the alcohol-induced reduction in the phosphorylation levels of Akt and GSK3β, including p-Akt308, p-Akt473, and p-GSK3β, indicating that IGFBP3 knockout promotes activation of the Akt/GSK3β signaling pathway (**Figures 6A–E**). This appears somewhat paradoxical when compared to our previous findings regarding IGFBP3 overexpression in AML12 cells, but it may be associated with elevated levels of free active IGF1. Analysis of the TMEM219/Caspase 8 signaling pathway revealed significant molecular changes in the hepatic tissues of ALD mice. Specifically, compared with their *Igfbp3^f/f^* littermates, *Alb-cre;Igfbp3^f/f^* mice exhibited a marked downregulation in the protein expression of both TMEM219 and Caspase-8 (**Figures 6A, F–H**). These findings suggest a previously uncharacterized crosstalk between the Akt/GSK3β and TMEM219/Caspase 8 pathways, which may involve compensatory signaling mechanisms that regulate apoptotic processes in alcoholic liver disease.

## 4 Discussion

In the present study, we performed a comprehensive analysis of the expression profiles of IGF system components in both cellular and animal models of ALD. To characterize the role of IGFBP3 in ALD pathogenesis, our results revealed a marked reduction in IGFBP3 expression during ALD progression, highlighting its central role in this disease process. Building on these observations, subsequent experiments demonstrated that overexpression of IGFBP3 significantly exacerbated ALD phenotypes in these models, characterized by increased alcohol-induced ROS production, lipid accumulation, and cellular apoptosis. In contrast, liver-specific knockout of IGFBP3 in *Alb-cre;Igfbp3^f/f^* mice attenuated alcohol-induced liver injury compared to *Igfbp3^f/f^* controls, underscoring the hepatoprotective effects of IGFBP3 ablation. To further dissect the IGF1-dependent and -independent functions of IGFBP3, we generated an IGFBP3 mutant variant (IGFBP3^GGG^) via site-directed mutagenesis, which specifically targets the IGF1-binding domain. Notably, overexpression of IGFBP3^GGG^ also exacerbated alcohol-induced liver injury, albeit with a relatively attenuated capacity to induce apoptosis compared to the IGFBP3 overexpression group. Furthermore, mechanistic investigations revealed that dysregulation of IGFBP3 expression disrupts the Akt/GSK3β and TMEM219/Caspase 8 signaling axes, thereby modulating the pathogenesis of ALD.

Akt serves as a pivotal mediator of signal transduction pathways triggered by growth factors, cytokines, metabolic hormones, and nutrients, and is recognized as a multifunctional kinase central to both physiological homeostasis and disease pathogenesis (Martelli et al., 2012). Previous studies have demonstrated that alcohol suppresses Akt expression both *in vitro* and *in vivo* (He et al., 2006; Zeng et al., 2018). However, other studies have presented seemingly contradictory findings, showing that alcohol activates the Akt signaling pathway (Zeng et al., 2012; Wang et al., 2024). This discrepancy may arise from differences in modeling approaches and variations in alcohol dosage administered. Our study demonstrated that exposure to 200 mM ethanol for 24 hours suppressed phosphorylation of Akt and its downstream target GSK3β in AML12 cells (**Figure 4**). A key mechanism linking ethanol to Akt inhibition involves CYP2E1 induction, which promotes oxidative stress and the generation of lipid peroxidation products such as 4-HNE, these metabolites can directly impair Akt/GSK3β signaling. IGF1 can mitigate this inhibition, highlighting a protective axis (Zeng et al., 2018). IGF-1 activates the Akt/GSK3β axis through the canonical PI3K–Akt signaling cascade: it binds to its receptor, leading to IRS phosphorylation, which in turn recruits and activates PI3K. Previous studies have shown that inhibiting this pathway with LY294002 (a PI3K inhibitor) blocks the ability of IGF-1 to counteract alcohol-induced metabolic disturbances in cells (He et al., 2006; Xu et al., 2018). Within this framework, IGFBP3 serves as a critical modulator of IGF1 bioavailability. Its downregulation during chronic alcohol exposure may elevate free IGF1 levels, thereby promoting Akt activation as a compensatory response. Conversely, IGFBP3 overexpression likely sequesters IGF1 and attenuates this protective signaling, exacerbating injury. In line with this model, our ELISA measurements of IGF-1 in the cell supernatant are consistent with these findings (**Figure 3C**). Notably, the increase in extracellular IGF-1 was observed only with wild-type IGFBP3 overexpression, but not with the IGFBP3^GGG^, indicating that IGFBP3 elevates detectable IGF-1 levels by stabilizing and prolonging IGF-1 half-life as a carrier protein, rather than by enhancing IGF-1 synthesis. Intriguingly, our study revealed that both overexpression of IGFBP3 and IGFBP3^GGG^ promoted alcohol-induced liver injury, with no significant difference in lipogenesis observed between the two groups (**Figure 3F**). These results are consistent with previous reports suggesting that IGFBP3 may exert IGF1-independent effects (Arab et al., 2020). Future studies incorporating direct assessment of IGF1R phosphorylation or targeted IGF1R inhibition will be required to conclusively dissect IGF1-dependent from IGF1-independent mechanisms.

Current research has shown that IGFBP3 can bind to the membrane receptor TMEM219 in an IGF1-independent manner, activating Caspase-8 signaling to promote apoptosis (Baxter, 2013). Consistent with this, our study demonstrated that overexpression of IGFBP3 increased the expression of TMEM219 and Caspase-8, thereby promoting alcohol-induced apoptosis (**Figures 3**, **4**). Recent studies have found that recombinant Chitinase-3-like protein 1 (Chi3l1) can activate the MAPK/Erk and Akt/PKB signaling pathways in lung epithelial cells and murine peritoneal macrophages via TMEM219, suggesting crosstalk between TMEM219 and Akt signaling (Lee et al., 2016). Additionally, a previous study reported that liver-specific knockout of Caspase-8 significantly alleviates steatosis and reduces hepatic triglyceride and free fatty acid contents (Hao et al., 2017). Collectively, these findings suggest that overexpression of IGFBP3 may promote alcohol-induced apoptosis and lipid accumulation through the TMEM219/Caspase-8 pathway. Notably, recent studies in the gastrointestinal tract have further established the IGFBP3/TMEM219 axis as a critical regulator of intestinal stem cell death and mucosal healing (Amabile et al., 2025; D'addio et al., 2025), supporting the broader pathophysiological relevance of this signaling pathway. However, definitive establishment of a causal role for TMEM219/Caspase-8 in mediating the effects of IGFBP3 in ALD awaits future interventional studies utilizing targeted loss-of-function approaches. Thus, our current data are correlative and do not prove causality.

Furthermore, our study demonstrated that liver-specific knockout of IGFBP3 alleviates alcohol-induced liver injury (**Figure 5**). Unexpectedly, knockout of IGFBP3 also increased the phosphorylation levels of Akt and GSK3β in ALD (**Figure 6**), which appears contradictory to our previous findings with IGFBP3 overexpression (**Figure 4**). In hepatocellular carcinoma (HCC) research, studies have revealed that p53 degradation triggers IGFBP3-dependent activation of the AKT/mTOR signaling pathway in an IGF1-dependent manner (Li et al., 2024). Whether a similar adaptive response occurs in the context of chronic liver injury remains unclear. Nonetheless, it is plausible that decreased IGFBP3 levels in ALD may represent a compensatory mechanism to preserve free IGF1 bioavailability and limit further hepatocellular damage. It should be noted that although our findings are consistent with previous reports showing decreased serum and hepatic levels of IGF1 and IGFBP3 in both rats after 14−week ethanol exposure and in clinical patients with alcoholic hepatitis (Lang et al., 2000; Huang et al., 2022), the models used in this study are designed to capture early hepatocellular injury, characterized by hepatic steatosis and mild inflammation (Ghosh Dastidar et al., 2018; Buyco et al., 2021). These acute models differ from the “acute−on−chronic” physiology and hepatic synthetic dysfunction typically observed in advanced clinical ALD. Therefore, the IGFBP3−dependent mechanisms identified here likely reflect its role in initial alcohol−induced stress rather than in late−stage alcoholic hepatitis. Future studies employing more severe or clinically relevant alcoholic hepatitis models will help clarify whether these pathways persist throughout disease progression. In conclusion, while our study identifies IGFBP3 as a mechanistic candidate in ALD, its therapeutic potential hinges on achieving liver-specific delivery to avoid systemic disruption of the IGF axis and metabolism, and on rigorous safety evaluation (Laron, 2001; Livingstone and Borai, 2014). Systemic modulation of IGFBP3 may have unintended effects on growth, metabolism, and cell survival in other tissues, representing a key limitation for clinical translation. Direct blocking experiments (e.g., using IGF1R or Akt inhibitors) are required to further dissect this paradoxical regulation. The relationship between the Akt and TMEM219 pathways downstream of IGFBP3 also remains to be elucidated.

## Data Availability

The original contributions presented in the study are included in the article/**Supplementary Material**. Further inquiries can be directed to the corresponding authors.
