# Supplementary material for: Knockout of IGFBP3 improves alcohol-induced liver injury via Akt/GSK3β and TMEM219/caspase 8 pathways
Source: Front Physiol. 2026 May 15;17:1775461. doi: 10.3389/fphys.2026.1775461 (PMC13218913; doi:10.3389/fphys.2026.1775461)

Supplementary figure- IGFBP3^{GGG} sequencing data

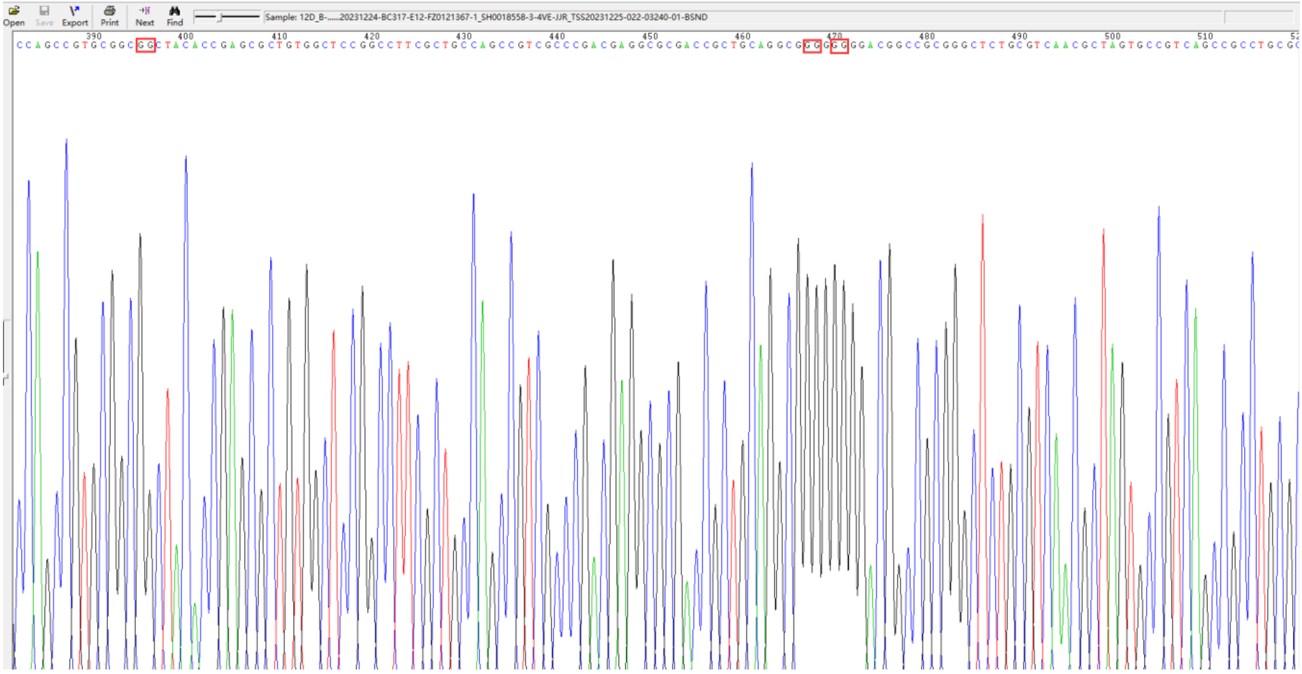

Supplementary figure-uncropped WB data

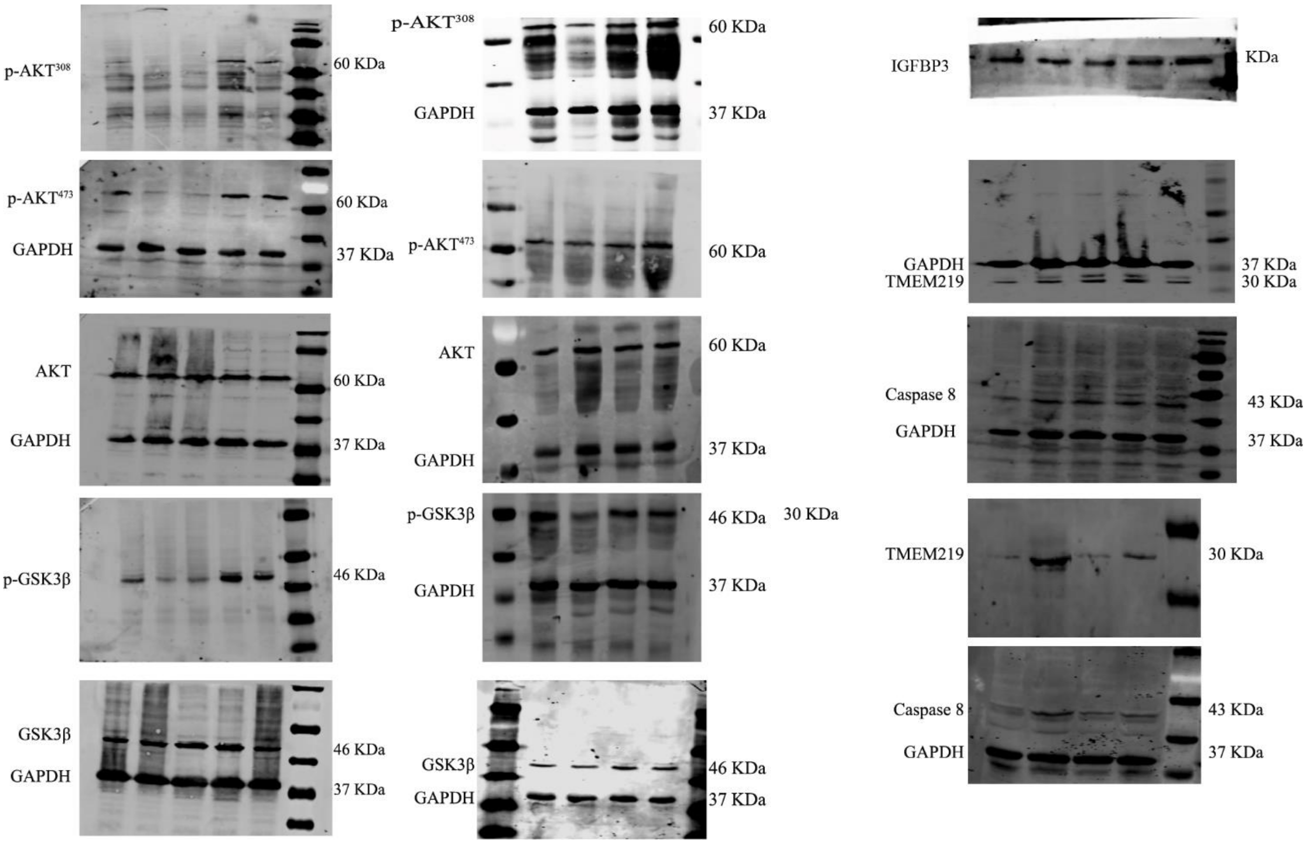

Supplement: Supplementary file 1 [file DataSheet1.pdf]
